# Effects of inter-individual variability in experimenters’ sensitivity and training on behavioral assessment of animal models of vestibular disorders

**DOI:** 10.3389/fneur.2025.1532927

**Published:** 2025-03-27

**Authors:** Romain Boularand, Bérénice Hatat, Claire Bringuier, Nicolas Chanut, Abdessadek El Ahmadi, Stéphane Besnard, Brahim Tighilet, Christian Chabbert

**Affiliations:** ^1^Vertidiag, Montpellier, France; ^2^Centre de Recherche en Psychologie et Neurosciences, Aix Marseille Université-CNRS UMR 7077, Marseille, France; ^3^Unité GDR 2074 CNRS, Marseille, France; ^4^UR Vertex 7480, Université de Caen Normandie, CHU Caen, Caen, France

**Keywords:** animal experimentation, risk of bias, methods, vestibular pathologies, inter-individual variability, pathological models

## Abstract

**Background:**

This study was designed to explore the correlation between animal behavioral assessment quality and rater’s individual sensitivity and training.

**Methods:**

We selected different raters to form a panel to rate the severity of posturo-locomotor deficits in animals displaying excitotoxic or ototoxic lesions-induced vestibular syndrome. All raters, regardless of their scientific level, received brief training based on videos and tutorial files. They then had to score videos of rats with different types and stages of vestibular syndromes. All data were collected and analyzed.

**Results:**

Inter-individual variability in raters significantly altered the results of behavioral assessment of posturo-locomotor deficits in vestibulo-lesioned animals. Neither gender nor scientific level had an impact on the results. In contrast, the sensitivity of the individual to animal welfare impacted the mean score in the ototoxic lesion model. Raters with high sensitivity tended to exaggerate the symptomatology.

**Conclusion:**

The use of automated assessments of posturo-locomotor deficits in vestibulo-lesioned rodents, is the best solution to limit these assessment biases.

## Highlights

- Identifying and reducing experimental bias is part of the scientific process to better control animal models predictability.
- Sensitivity of raters to animal welfare impacts behavioral assessment in ototoxic lesion model.
- Small raters team with similar training can avoid bias linked to inter-individual variability.

## Introduction

Animal pathological models aim to reproduce the biological, physiological and behavioral correlates found in human diseases. The main added value is to offer the possibility to investigate the cellular effects, as well as to screen and test therapeutic targets. They also allow precise monitoring of the course of the pathology and prediction of its consequences for human health. In neurotology as in other medical fields, improvement of the predictability of sensory vestibular damage models is a challenge that researchers are trying to meet (1) by combining biological, physiological and behavioral markers that should be quantitatively scored in a homogeneous manner.

Identifying and reducing the various experimental biases is part of the scientific process to best control this predictability, but also to guaranty reproductivity of data between and within laboratories (2–4). The research community is becoming increasingly vigilant regarding the possible impact of current scientific practices on the reliability and validity of published research. Besides essential data such as animal sex or age, which may greatly affect biological measures, and are not always mentioned (5), it is now known that even subtle changes in housing (cage size, social isolation or enrichment, noise pollution) (6, 7), training condition (especially positive reinforcement training) and the sequence of experiments, can also significantly alter the expression of symptoms of induced pathology and its recovery processes (8, 9) These observations have led to a range of rules regarding housing and experimental conditions that together may help to ensure animal welfare (10, 11).

In addition to these conditions directly affecting the animals, parameters specific to the experimenters, referred to as experimenter effects (12), or experimenter bias (13), can also be a source of variation in obtained results in the behavioral monitoring of animals in experimentation. This problem has been reported in a few behavioral studies involving rodents that were not automated and had to be rated by different experimenters. In these studies, the scoring of animals in elevated plus maze significantly differed depending on the experimenter (14).

Some of this bias arises from the experimenters’ judgment regarding behaviors. Experience and training are therefore essential for conducting behavioral experiments, because failure to consider essential factors affecting the behavior of animals, the interaction of animals and experimenters (15), and scoring behavior, may strongly influence the reproducibility, validity and reliability of the experiments (16–20). Other studies in rodents have reported bias related to the gender of experimenters (21) with for example a modulation of antidepressant-like effect of ketamine (22) or an impact of male-related odors that significantly altered ratings of pain exhibited by mice (23). In the case of animal models of vestibular pathologies with strong clinical disturbances, the experimenter bias in the assessment of behavioral biomarkers of the vestibulopathy has never been explored. It is precisely to address this issue that this study was designed.

We conducted a behavioral follow-up study of rats with excitotoxic or ototoxic vestibular lesions, under conditions similar to those used in our previous studies in terms of choice of animals, housing, handling sequences and observation methods (24–26). Both models evoked a panel of posturo-locomotor symptoms including immobility, inability to stand up on the hind limb, impaired posture and walk, head tilt, retropulsion, circling, vertical repetitive movements of the head. Each of these individual symptom varies with their own kinetics (27).

We selected different experimenters to form a panel to rate the intensity of posturo-locomotor deficits in vestibulo-injured animals. Experimenters were themselves assessed regarding their sensitivity to animal welfare, which relies to the individual perception of the animal suffering upon their use or exploitation such as scientific experimentation, and their level of training in the behavioral assessment of posturo-locomotor symptoms.

A study was then performed to search for correlation between raters’ inter-individual characteristics and animal scoring. The results of this study and their consequences in term of potential experimental bias are discussed.

## Materials and methods

### General procedures

Male Long Evans rats (8–10 weeks, 275 ± 25 g, Janvier, France) were housed in cages in social groups (4 rats per cages) with enrichment of the environment under constant temperature (20 ± 1°C), humidity (60 ± 5%) and brightness (below 110 Lux) conditions. Rats were kept on a light / darkness cycle of 12 h / 12 h (lights on at 7.00 am) with food and water available ad libitum. The animals were acclimatized to the experimenter and the experiment room for 5 days before the beginning of the experiments. The pre-defined endpoints were monitored throughout the study.

All animal experiments complied with the ARRIVE guidelines and are carried out in accordance with the U.K. Animals (Scientific Procedures) Act, 1986 and associated guidelines, EU Directive 2010/63/EU for animal experiments, or the National Research Council’s Guide for the Care and Use of Laboratory Animals under the veterinary and National Ethical Committee supervision (French Agriculture Ministry Authorization nF34-172-05). All the applied procedures were previously approved by the ethics committee (MP-CEPAL n°22).

### Assessment of vestibular syndrome

#### Vestibular lesion procedure

Animals were anesthetized with isoflurane®. Then, induction of the unilateral excitotoxic-type vestibular injury was carried out by a transtympanic administration of 100 μL of 25 mM Kainic acid (TTK) in 0.9% NaCl or of 25 mM sodium Arsanilate (TTA) in the left ear. A subcutaneous injection of 1 mL of sterile 0.9% NaCl prevented dehydration in rats. They were kept under anesthesia in a lateral position for additional 30 min (28–30) to allow the administrated compound to bathe the middle ear windows. Animals remained isolated for 24 h after surgery and were then put back in a collective cage. The general condition of the rat, weight and pre-defined endpoints were monitored throughout the operative process and post-operative follow-up.

#### Experimental design

An assessment panel composed of 8 volunteers and 4 members of the team was set up to determine whether there was any impact of the characteristics of the experimenters on the scoring of animals and the results of a study. Each member of the panel received training through 1 tutorial video of 5 min and 1 descriptive sheet per lesion model (TTK and TTA). They then had to score the severity of vestibular syndrome through 26 videos of TTK rats and 25 videos of TTA rats covering all post-lesionnal time points before and after the TT injections. The test was performed a first time 3 days before a left trans-tympanic ear injection to obtain a pre-operative reference value, then at different times after injection (2 h, 4 h, 1, 2, 3, 7 and 14 days). All videos had an identical length of 2 min. Panel members and data analysts were only aware of the experimental model of the animal in videos, while the time point of the video recording remained unknown. We aimed here to evaluate the sensitivity of the experimenters to the different intensities of the vestibular syndrome expression. The syndrome presents a peak expression in the first hours after the lesion, then gradually decreases over time with the impact of the vestibular compensation. The videos evaluated by the raters presented acute and compensated phases of the syndrome. The aim was to assess up to what intensity an experimenter was able to detect a symptom and score it, whatever the observation delay. Finally, they answered a questionnaire on the characteristics and amplitudes of the posturo-locomotor symptoms (Appendix).

#### Vestibular syndrome rating

The vestibular syndrome was first activated by elevating the rats by the tail about 20 centimeters from the support to activate the vestibular syndrome and exacerbate the expression of its symptoms. It was assessed for 2 min and was composed of a typical set of posture locomotor symptoms that we were able to determine on the basis of our experience. Those symptoms were together present in the acute phase and disappeared sequentially after a specific time (31). A score of 0 (no symptom) to 3 (severe symptoms) was assigned to the following 10 criteria for the TTK model and 8 for the TTA model for a maximum total score of 30 or 24 respectively: prostration time (immobility), ability to stand up on the hind limb (rearing), quality of gait and general movements of the animal (quality of locomotion), body height and lift (body height), lateral tilt of the head (head tilt), reverse walk (retropulsion), concentric trajectories (circling), vertical repetitive movements of the head (bobbing) and general condition. For the TTK model only, we assessed torsional movement of the body when the rat is lifted by the tail over the support (tail hanging behavior), and landing reflex of the rat when dropped on the support (landing reflex).

### Panel characteristics

#### Panel recruitment

Panel recruitment was done on a voluntary basis with persons accustomed or not to animal experimentation. The final panel was composed of 8 volunteers (lab students and staff who were not involved in animal experiment) and 4 members of the team, with 7 women and 5 men. Only the 4 members of the team were previously trained in vestibular syndrome scoring on animal model. The sensitivity to animal welfare can be defined as the way in which we collectively or individually view the way in which animals are regarded and treated in human societies.

#### Panel formation

Panel members were not used to scoring animals with vestibular lesion, so they had to be trained. Each member of the panel received training through one tutorial video of 5 min and one descriptive sheet per lesion model (TTK and TTA). They were also given a questionnaire to complete about their previous training, the quality of their training, and their ability to self-assess rat symptoms.

### Behavioral tests

The impact of vestibular syndrome on the overall motor and exploratory behavior of the animal was assessed using the Open field test. The animals were accustomed to the device for 5 min per day for 3 days before the start of the experimental follow-up. The vestibular syndrome was first stimulated by elevating the rats by the tail about 20 centimeters from the support in order to exacerbate the expression of symptoms.

Rats with Kainic acid administration were tested on a white neutral and empty surface. Animal were placed in the center of this area and allowed to explore. Their behavior was recorded for 2 min with a webcam placed on the roof. The test was performed a first time 3 days before a left trans-tympanic ear injection to obtain a pre-operative reference value, then at different times after injection (2 h, 4 h, 1, 2, 3, 7 and 14 days).

Rat with Arsanilate injection were tested in an open field. The open field was a white neutral and empty surface of 80x80x40cm. Animals were placed in the center of the field considered more stressful compared to the periphery, and their behavior was recorded for 5 min using a digital camera with the Software Ethovision™ XT 15 (Noldus). A phase of habituation to the environment was allowed. The test was performed a first time 3 days before a left trans-tympanic ear injection to obtain a pre-operative reference value, then at different times after the injection (1, 2, 3, 7, 14 and 21 days). The light was fixed at 50 Lux in the center and 30 Lux in corners of the maze. Walls were thoroughly cleaned between animals with ethanol solution (20%) (20). A member of the team randomly chose videos at each time point, including pre-operative, which were submitted to the panel for assessment.

### Statistical analysis

The primary objective of our manuscript is to evaluate the performance of raters in detecting differences (if any) between symptoms and animals. The study is designed around the crossing of three experimental factors: Raters, Animals, and Symptoms, with an additional factor, Sessions. This type of experimental design is relatively common in other disciplines, particularly sensory analysis, which focuses on evaluating assessors’ ability to discriminate between products using various descriptors or attributes (32). For our statistical analysis, we followed standard practices in sensory analysis, as outlined by Per Lea and colleagues (33). Specifically, we employed the following ANOVA model: responses ~ Raters * Animals * Symptoms * Sessions.

This model allows us to account for the effects of individual factors as well as their interactions. In this framework, the Raters and Animals factors are considered random effects, while Symptoms and Sessions are treated as fixed factors. This approach enables us to address several key questions: Are the raters effective in their evaluations? Can they reliably discriminate between different symptoms? Are their assessments consistent across repeated evaluations? Do their evaluations align with those of the panel as a whole? To perform this analysis, we first used the panelperf function from the SensoMineR package in the statistical software R (see https://cran.r-project.org/web/packages/SensoMineR/SensoMineR.pdf). Additionally, we utilized the JASP and JAMOVI software tools to perform complementary analyses. Beyond the ANOVA approach, we also applied the Rasch model (34), which models a rater’s response to a symptom as a function of the difference between the rater’s ability, and the characteristics of the symptom. This model provides a visual representation of the distribution of raters’ abilities in relation to the characteristics of symptoms (specifically, their “accessibility”). Such a representation offers valuable insights into the discriminatory power of raters and the inherent difficulty of the symptoms being assessed. Values were expressed as mean ± SEM. A significant statistical result is indicated by ^*^ if *p* value < 0.05, ^**^ if *p* value < 0.01, and ^***^ if *p* value < 0.001.

## Results

**Figure 1:**
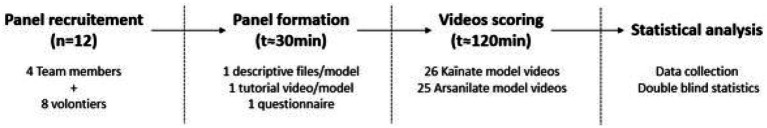
Experimental design.

A questionnaire on scientific experience, experience in animal study, animal scoring and sensitivity to animal welfare was sent to each member of the experimenters panel. We grouped the experimenters according to different parameters. The questionnaire provided an overview of the heterogeneity of the selected experimenters and their specificities (Table 1). The experimenter panel was composed 58% of women and 42% of men with an average age of 30 years (± 8 years). Having chosen a largely scientific panel with 25% experienced and 75% very experienced, but not accustomed to animal studies (42% non-experienced), we also questioned their sensitivity to animal welfare. The questionnaire revealed that 18% were non-sensitive, 50% were sensitive and 42% were very sensitive. More broadly, in our sample, people experienced in animal studies (58%) were not necessarily qualified (33% experienced) for scoring posturo-locomotor deficits in vestibulo-injured animals (Figure 1).

**Table 1:** Panel overview based on the questionnaire given in Appendix.

Panel gender	58% Women	42% Men	
Age mean	30 years (± 8 years)		
Animal cause sensibility	8% Non-sensitive	50% Sensitive	42% Very sensitive
Scientific experience	0% Non-experienced	25% Experienced	75% Very experienced
Animal study experience	42% Non-experienced	25% experienced	33% Very experienced
Animal scoring experience	67% Non-experienced	8% Experienced	25% Very experienced

Using the Rasch model assessment of our questionnaire, we found that some items, such as rearing and retropulsion, were similarly perceived (easy or difficult to score, respectively) in both the in TTK- and TTA-induced vestibulopathy models. Other items were diversely appreciated in the two models: circling, head tilt and bobbing were easy to assess in the TTK model, whereas they were considered difficult to score in the TTA model. Conversely, immobility was easy to assess in the TTA model, whereas it was considered difficult to score in the TTK model (Table 2). These data indicate that in the present study, difficulties in scoring the considered symptom were model-dependent.

**Table 2:** Assessment of the difficulties in scoring a symptom in vestibular loss model with the Rasch model.

Model	Tail hanging	Immobility	Displacement	Landing reflex	Rearing	Bobbing	Overall health	Head tilt	Retropulsion	Circling
TTK	−0.21	−1.63	−0.097	0.23	−1.51	1.16	−0.104	1.92	2.41	2.47
TTA		1.46	−0.62		−2.28	−0.23	−0.37	−0.14	1.83	0.34

This is a mathematical approach developed by Georg Rasch for analyzing answers to questions on a reading assessment or questionnaire responses, as a function of the trade-off between the respondent’s abilities, attitudes or personality traits. This approach specifically defines difficulty/facility as the sole parameter of interest when assessing items. The more negative the element, the easier it is to score it. Conversely, the more positive the item is, harder it is to score it.

**Figure 2:**
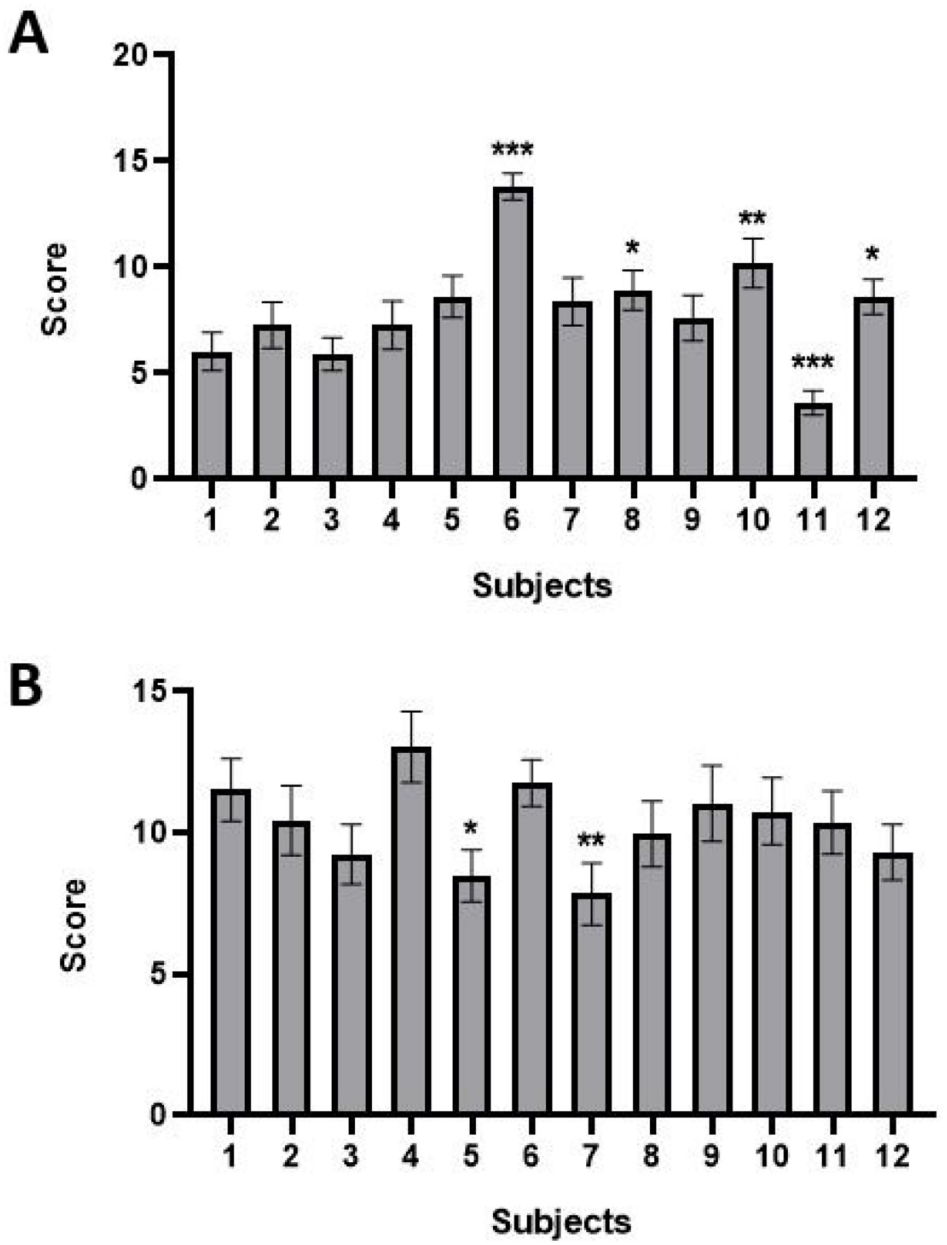
Impact of intra-individual variability on average rating of vestibular syndrome induced by an injection of Kainate **(A)** and Arsanilate **(B)**. ANOVA 2 factors, Subject * Score, with repeated measures, *p* value <0.001 and a t test to compare sample with a hypothetical mean. Significant statistical result are indicated by ^*^ if *p* value < 0.05, ^**^ if *p* value < 0.01, and ^***^ if *p* value < 0.001.

Subjective scoring makes it possible to assess the severity of vestibular syndrome by scoring different symptoms using four grades from 0 to 3 (Figure 2). The 12 members of the panel assessed videos of rats that received a trans-tympanic administration of either kainic or arsenic acids and the average scores were reported. We found significant differences in the way the vestibular disorders symptoms were scored between raters in the two experimental models (*p* < 0.001). A one sample *t* test was performed to compare every rater with a hypothetical mean resulting from the mean of all raters (Figures 2A,B).

**Figure 3:**
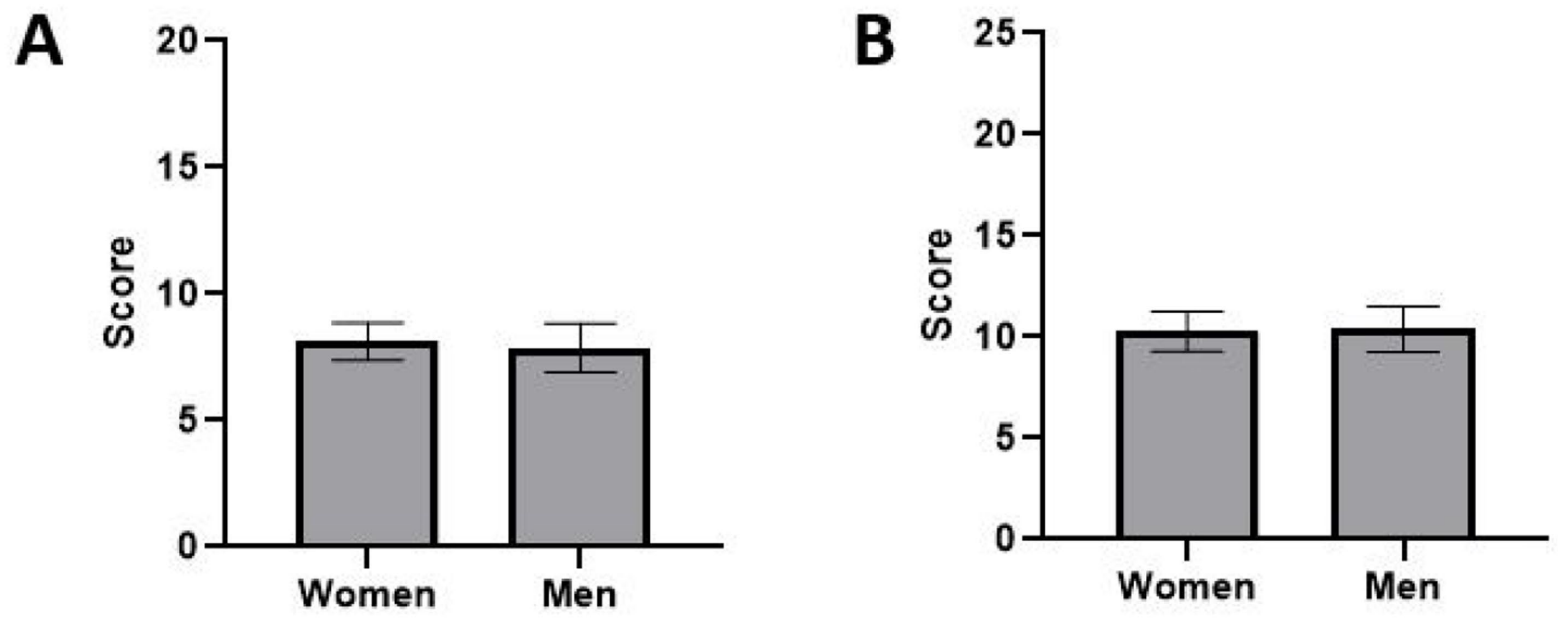
Differences in the average rating of vestibular syndrome according to gender of experimenters on the two experimental models **(A)** TTK and **(B)** TTA. ANOVA 2 factors, Gender * Score.

The questionnaire enabled classification of the different members of the raters panel according to their characteristics. Thus, we could assess whether one of the parameters of individuals had a role in the variability in the average symptoms score (Figure 3). No gender difference of raters was observed between both TTK and TTA models (*p* = 0.59) (Figures 3A,B).

**Figure 4:**
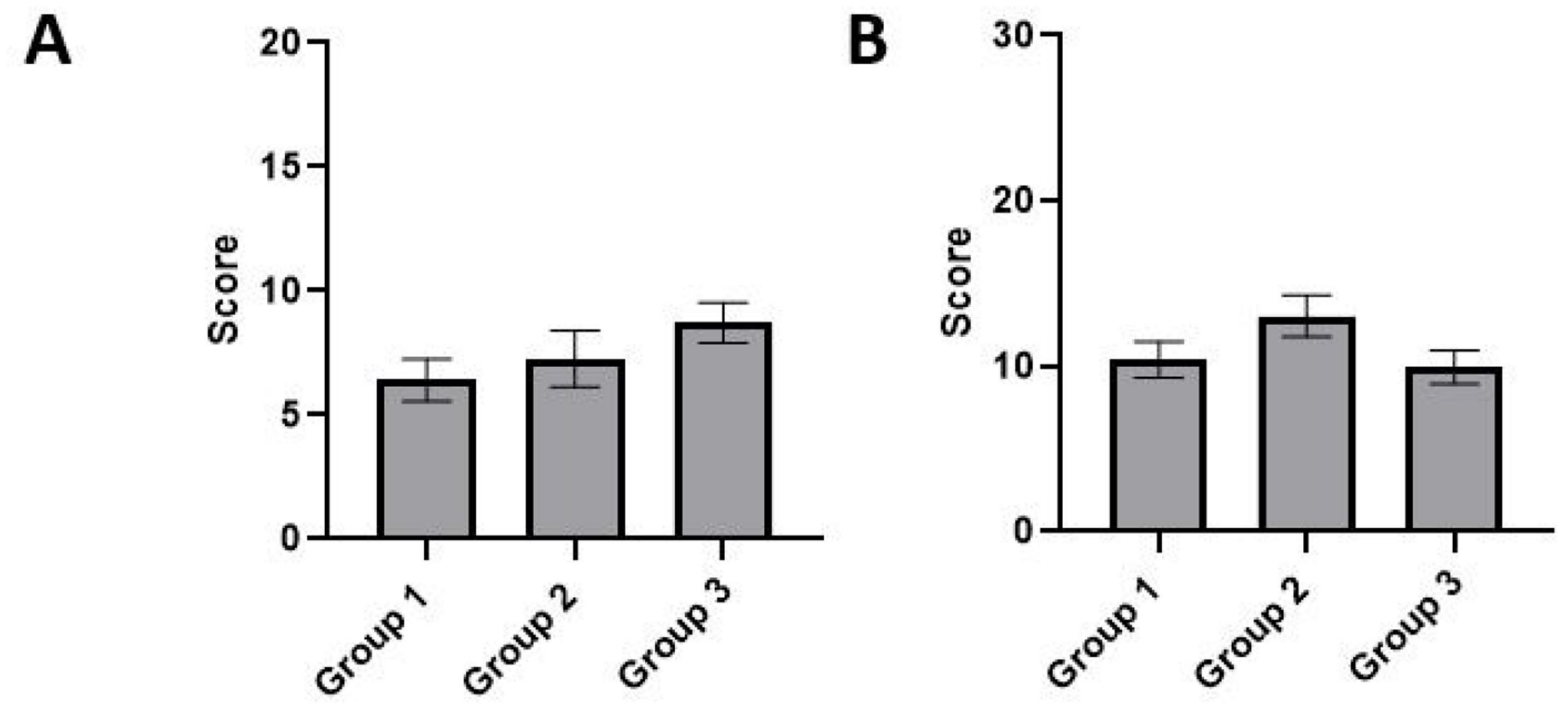
Differences in the average rating of vestibular syndrome according to the experience with animals of raters on two lesion models. There were 3 different groups: Group 1: Non-experienced (*n* = 5), Group 2: Experienced (*n* = 3), Group 3: Very experienced (*n* = 4). Two types of models were studied **(A)** TTK and **(B)** TTA. ANOVA 2 factors, Experience.

Panel members were regrouped in 3 different groups according to their experience (Group 1: non-experienced; Group 2: experienced; Group 3: very experienced; Table 1). We did not find statistically significant differences in the average rating between the 3 groups in both the TTK (*p* = 0.93) (Figure 4A) and TTA (*p* = 0.22) models (Figure 4B).

**Figure 5:**
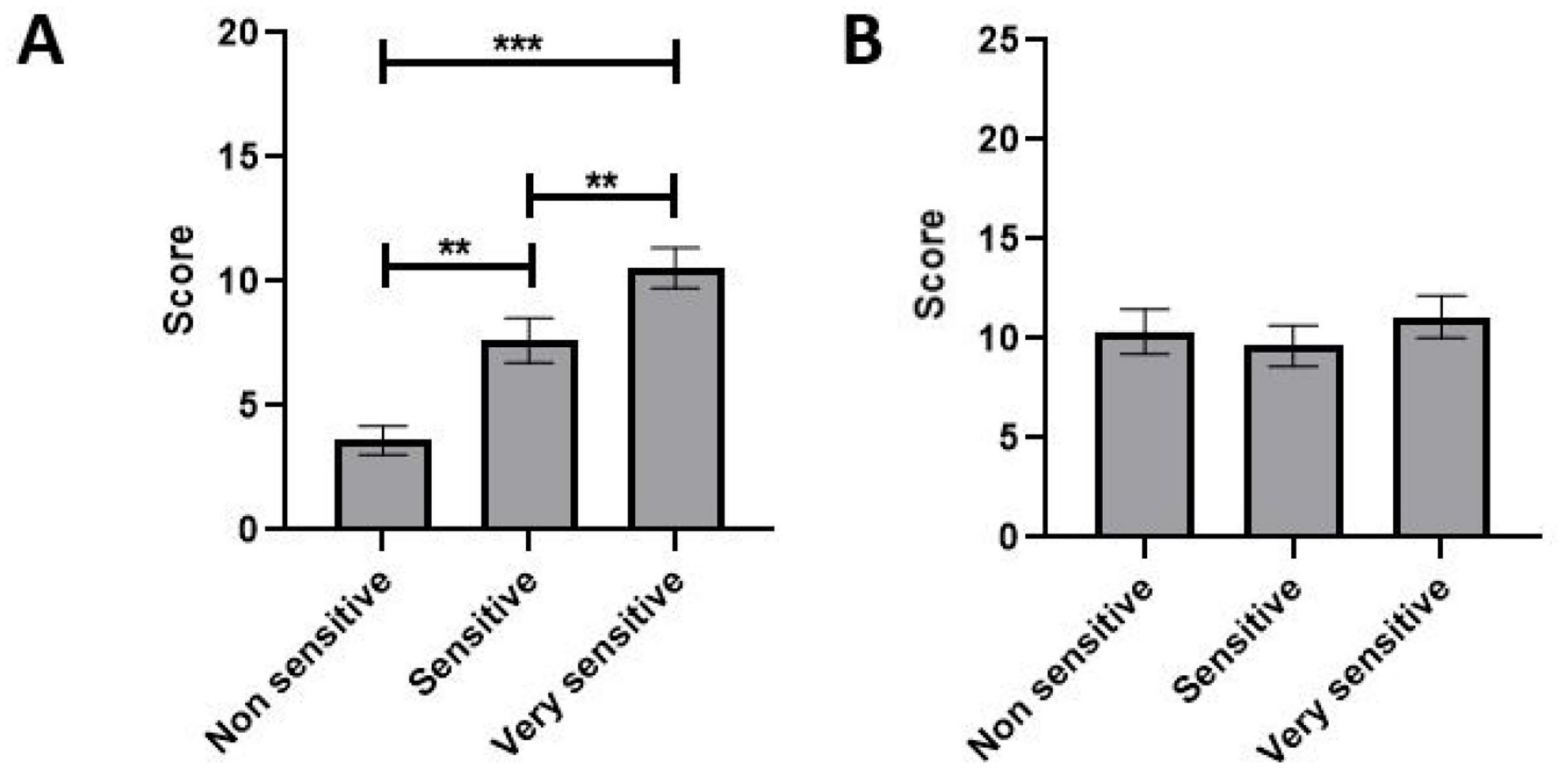
Differences in the average rating of vestibular syndrome according to the sensitivity to animal welfare of raters on two lesion models. Group 1: non sensitive (*n* = 1), Group 2: sensitive (*n* = 6), Group 3: very sensitive (*n* = 5). **(A)** TTK and **(B)** TTA. ANOVA 2 factors, significant statistical result are indicated by ^**^ if *p* value < 0.01, and ^***^ if *p* value < 0.001.

Another interesting characteristic is the sensitivity to animal welfare of the experimenters. There is a range of sensitivity in the panel from non-sensitive to very sensitive (Table 1). We found that the sensitivity has a significant impact on the average rating of vestibular syndrome in the TTK model (*p* = 0.02) (Figure 5A). With a posthoc test of Tukey, it appears that the non-sensitive group was significantly different from the sensitive group (*p* value <0.001) and the very sensitive group (p value <0.001). Likewise, the sensitive group was significantly different than the very sensitive group (*p* < 0.001). In contrast, the sensitivity had no significant impact on the average scoring of the TTA model (*p* = 0.84) (Figure 5B).

**Figure 6:**
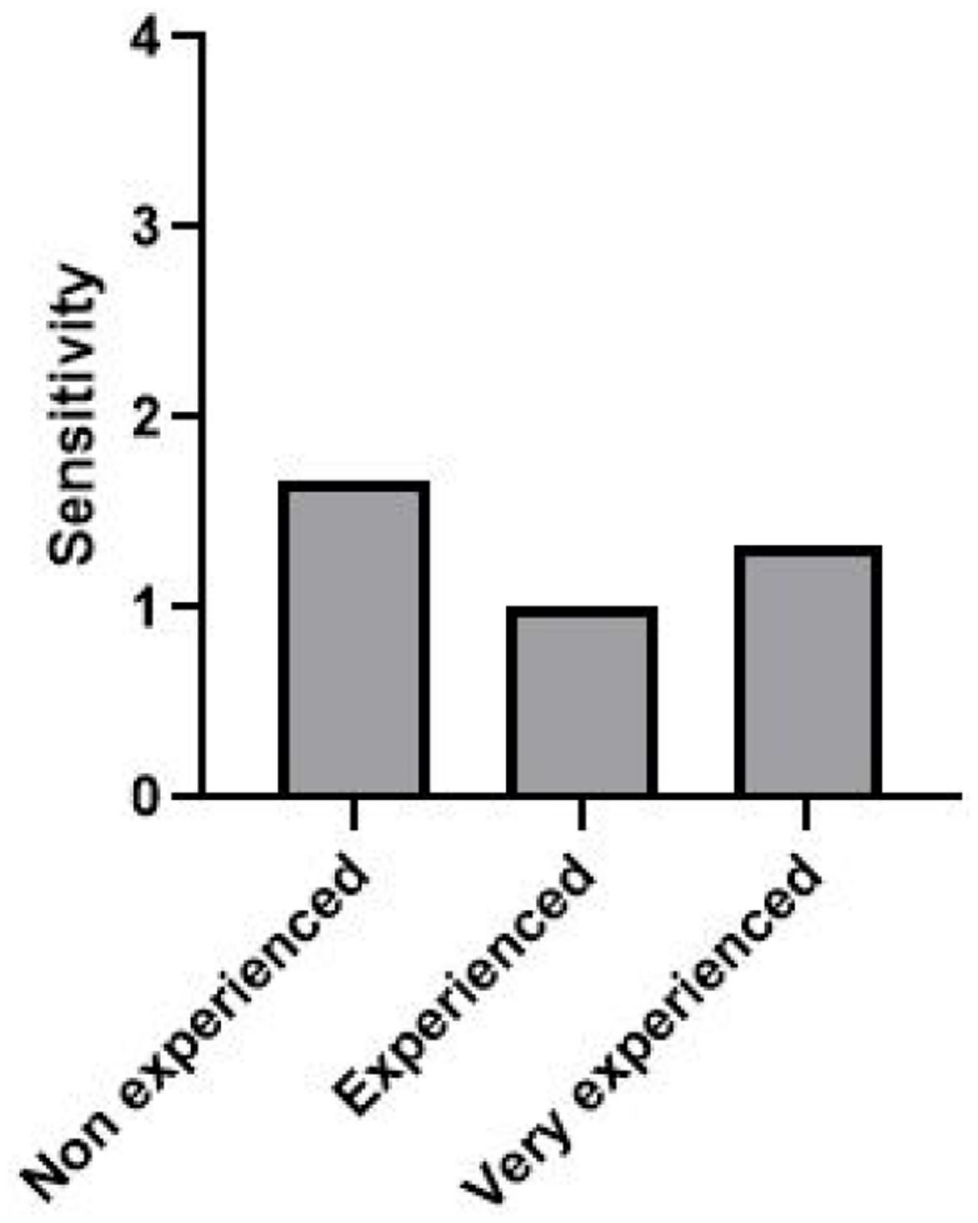
Differences in the sensitivity to animal welfare depending on their experience in animal study. ANOVA 2 factors, Sensitivity * Experience. Non-experienced (*n* = 5), Group 2: Experienced (*n* = 3), Group 3: Very experienced (*n* = 4).

There was no significant evidence of a link between the sensitivity to animal welfare and the experience in animal study in the panel (*p* = 0.64) (Figure 6).

**Figure 7:**
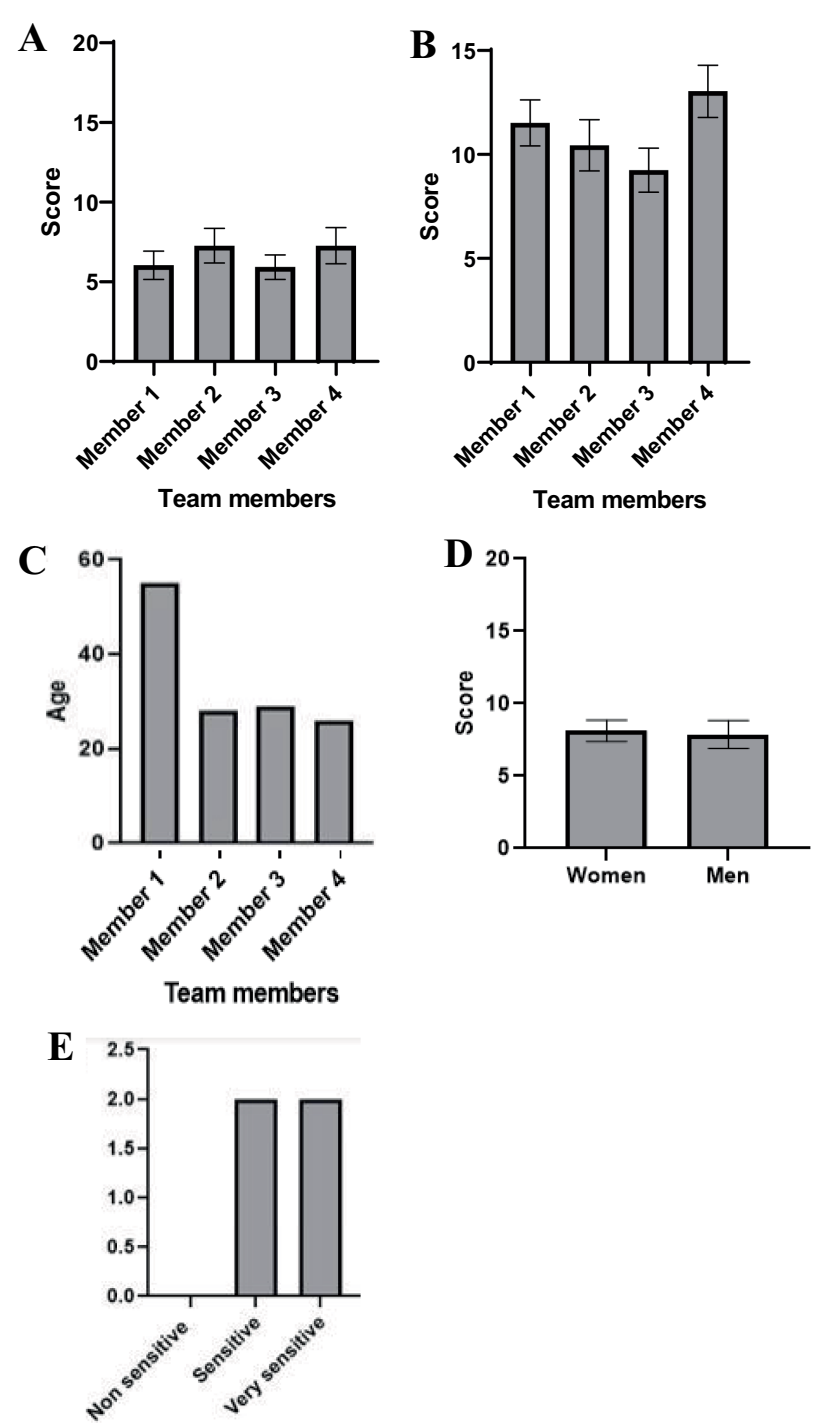
Impact of equal and long-term training on average rating of vestibular syndrome induced by an injection of Kainate **(A)** and Arsanilate **(B)** considering gender, age and sensitivity difference. ANOVA 2 factors, Subject * Score, with repeated measures, *p* value = 0.63 for TTK model and *p* value = 0.13 for TTA model. Descriptive analysis of team members with their age **(C)**, gender **(D)** and sensitivity to animal welfare **(E)**.

Interestingly, no significant differences were found in the way vestibular disorders symptoms were scored between raters who are team members (*p* = 0.63 for TTK model and *p* = 0.13 for TTA model, Figures 7A,B), even if there are differences in age or gender (Figures 7C,D), and more surprisingly even if they show differences in sensitivity to animal welfare (Figure 7E).

## Discussion

### Identify biases that can occur in an animal study to avoid erroneous conclusions

Evidence of bias in animal studies was previously shown in published research (10, 11). Arguably, the degree to which environmental variables influence research outcomes may not be considered by many researchers (35–37) when they focus on controlling direct experimental variables inherent to their own research. The ultimate research facility, with specialized housing rooms and caging systems, provides an environment where variables are minimized regardless of species, thus creating an optimal setting and preventing bias in the data. Animal facilities specificities can account for major differences between studies (4). However, the differences between scientists who are in contact with animals also subsist. Notably, when rating scales are used, some significant differences can occur. We have tried to identify key points to keep in mind when this type of scoring is used.

We found a significant experimenter effect on the average score of TTK and TTA models which means that one experimenter can probably overstate an effect while another can minimize or even miss it.

#### Gender

We did not find any evidence of an influence of gender on the ability to score the vestibular disorders symptoms. Bias depending on gender was previously reported in behavioral assessments of posturo-locomotor and cognitive abilities in mice models of ethanol effects (18), and assessments of pain in mice (23). In the first case, olfactory exposure to male experimenters’ odors significantly altered the ability of mice to maintain their balance on a rotarod and cognitive parameters assessed in elevated plus maze or open field. In the second case, olfactory exposure to human male odors induced stress and related analgesia in the rodents. These effects may be triggered by male odors in outfits worn by experimenters on the previous day, or specific chemicals known to be excreted by men such as 3-methyl-2-hexenoic acid, androsterone, or androstadienone. In the present study, this situation could not be studied as ratings were performed posteriori, without contact with animals.

#### Experience with animals

Surprisingly, experience of raters in scoring posturo-locomotor symptoms in both the TTK and TTA rat models did not significantly influence their ratings. One might have thought that experience would be a determining factor in the detection and quantification of such specific symptoms, as experience with laboratory animals has been demonstrated to significantly influence how behavioral markers of animal suffering are accounted for (38). It seems that the behavioral parameters measured are sufficiently salient for novice experimenters to obtain similar average scores to specialist raters. This observation further validates the predictability of the animal models of peripheral vestibular disorders used in present study (1).

#### Sensitivity to animal welfare

This has become a major societal issue in recent years, and is the basis for animal welfare vigilance in both research and industry. This issue raises major cultural, economic and ecological issues and has led to legislative developments in Western countries in particular, with legal recognition that animals are sentient beings, which requires them to be treated appropriately. The sensitivity to animal welfare varies greatly among individuals, depending on their own sensitivity, experience and contact with animals. Based on our assessments, the sensitivity to animal welfare appears to be the main parameter which supports the experimenter effect on the scoring of the TTK model. This parameter does not significantly affect the score in the TTA model. This can be tentatively explained by the fact that the less severe injury models (such as the TTK model) leave more room for interpretation than the more severe models (such as the TTA model). Less animal-sensitive subjects will thus tend to underestimate the vestibular syndrome compared to those who are more sensitive to animal welfare. Although the evaluation of animal experimentation videos is independent of the experimenter questionnaire, we cannot rule out the idea that having started the questionnaire with the question of sensitivity to the animal cause may have further sensitized the experimenter who consider themselves to be sensitive. Hence being a source of potential bias in the design.

### Anticipation of the scoring scale and training of raters to smooth the results

The behavioral scales used in the TTK (24) and TTA (25, 26) models contain items that are not easy to assess for people who are not accustomed to animal experiments. Thus, this margin of error in the symptoms quantification may lead to a significant experimenter bias. We note that in the team the average rating is very close when comparing all raters. Thus, the team having been trained identically for 2 months with different sensitivity in animal welfare at the beginning, that appears in our study as a major parameter impacting the TTK model scoring, now show a reproducible rating with perfect homogeneity between raters. This means that working on the standardization (39) of a scoring scale with adaptive items when subjects are inexperienced or implementing long training with the team and animals makes it possible to smooth the results and arrive at exploitable and reproducible conclusions even if there is intra-individual variation at the beginning.

Automated behavioral assessment is undoubtedly a solution to avoid biases reported in this study. As an example, videotracking and dynamic weight bearing device have been demonstrated to be a suitable tools to monitor specific posturo-locomotor deficits following unilateral vestibular insult in rodents (40–42). However, it is not always available in all research teams. Many of which still use subjective behavioral assessment. It’s even less the case in human vestibular rehabilitation practices. Present study aimed to emphasize the risks of bias in subjective assessment.

## Conclusion

This study is to our knowledge the first to identify and assess potential experimenter bias in the behavioral assessment of postural-locomotor symptoms in rodent models of peripheral vestibulopathies. The results of our investigations show that while the gender of the experimenters and their experience with animals have little influence on the way the scoring is performed, sensitivity to animal welfare is a potential source of experimenter bias. Training of experimenters in animal behavioral assessment procedures can reduce the risk of experimenter bias. This should be considered in non-automated behavioral assessment studies.

## Data Availability

The raw data supporting the conclusions of this article will be made available by the authors, without undue reservation.

## Appendix

Questionnaire

Name of the experimenter:

Gender:

Age:

Do you know one or more people with vestibular disorders in your entourage?:

Highlight the correct answer:

Sensitivity to the animal cause: 0 (non-sensitive) – 1 (sensitive) – 2 (very sensitive)

Experience in the scientific field: 0 (no experience) – 1 (some experience) – 2 (very experienced)

Experience in animal experimentation (rodents): 0 (no experience) – 1 (some experience) – 2 (very experienced)

Experience in subjective scoring: 0 (no experience) – 1 (some experience) – 2 (very experienced)

Experience in subjective TTA scoring: 0 (no experience) – 1 (some experience) – 2 (very experienced)

Experience in subjective TTK scoring: 0 (no experience) – 1 (some experience) – 2 (very experienced)

For subjective scoring to be reliable, estimate the level that the experimenter would need: 0 (Beginner level) – 1 (Amateur level) – 2 (Intermediate level) – 3 (Experienced level) – 2 (Very experienced level)

Would you have liked more complete training in order to feel more confident with your scoring?: Yes/No

If so, what did you lack?:

- A more detailed information sheet describing the experiment: Yes/No
- More tutorial videos (details on each item): Yes/No
- A Skype interview with an experienced trainer: Yes/No
- A brief training session with animals in the field: Yes/No
